# Supplementary material for: WordCommentsAnalyzer: A windows software tool for qualitative research
Source: F1000Res. 2018 Sep 4;7:536. Originally published 2018 May 3. [Version 2] doi: 10.12688/f1000research.14819.2 (PMC6137411; doi:10.12688/f1000research.14819.2)
Supplement: Supplementary file 3 [file f1000research-7-17614-s0002.tgz › b2a79496-4ada-47c4-9682-a99ce867836d.zip!/Eight Iranian Health Journals/Journal of Health System Research _20.docx]

© Journal of Health System Research . This work is licensed under CC BY-NC 4.0 (https://creativecommons.org/licenses/by-nc/4.0/).

Volume & Issue: دوره 9، شماره 12؛ 1392

Title: برآورد انرژی مصرفی حین انجام کار به روش برآورد سیستماتیک بار کار (SWE) در کارگران مرد یک کارخانه تولید سازه های فلزی

http://hsr.mui.ac.ir/index.php/jhsr/article/view/1153

مقدمه: در زمینه تناسب و تطابق کار با کارگر، دانستن بار کاری قابل قبول از اهمیت بالایی بر خوردار است. این مطالعه با هدف برآورد انرژی مصرفی حین انجام کار و تعیین عوامل موثر بر آن در کارگران یک کارخانه تولید سازه‌های فلزی صورت پذیرفت. روش‌ها: در این مطالعه مقطعی تمامی کارگران کارخانه فولاد پایه فارس که دارای کار دینامیک بودند (100 نفر) مورد ارزیابی قرار گرفتند. بخشی از داده‌ها به وسیله پرسشنامه و بخشی دیگر از طریق چک لیست برآورد سیستماتیک بار کار (SWE) گردآوری شد. پرسشنامه مورد استفاده خود از دو قسمت تشکیل شده که بخش اول ویژگی‌های دموگرافیک و بخش دوم وپژگی‌های آنتروپومتریک و فیزیولوژیک فرد را شامل می‌شد. یافته‌ها: میانگین و انحراف استاندارد سن در کارگران مورد مطالعه به ترتیب برابر با 84/30 و 59/6 سال به دست آمد. انرژی مصرفی حین انجام کار در کارگران مورد مطالعه برابر با 809/0±81/2 کیلوکالری در دقیقه برآورد شد. یافته‌های این مطالعه نشان داد که بین انرژی مصرفی افراد حین انجام کار (کیلو کالری در دقیقه) و ضربان قلب در حالت استراحت و حین کار رابطه معنی‌داری وجود دارد. همچنین نتایج این مطالعه نشان داد که بین انرژی مصرفی و قد، وزن و نمایه توده بدن ارتباط معنی‌داری وجود دارد، در صورتی که بین انرژی مصرفی حین انجام کار و سن و سابقه کار ارتباط معنی‌داری مشاهده نشد. نتیجه‌گیری: از متغیرهایی همچون ضربان قلب حین استراحت و حین کار، قد، وزن و نمایه توده بدن می‌توان به عنوان عوامل موثر بر انرژی مصرفی حین انجام کار نام برد.

Title: ارزیابی وضعیت آسایش حرارتی کارکنان مرکز آموزشی درمانی الزهرا(س) و مقایسه آن با استاندارد اشری

http://hsr.mui.ac.ir/index.php/jhsr/article/view/1454

مقدمه: امروزه بحث آسایش حرارتی با توجه به پیشرفت تکنولوژی و کاربرد تجهیزات و وسایل مختلف در محیط کار براي مدیران سازمان ها و نهاد های دولتی و غیر دولتی در محیط های صنعتی و غیر صنعتی بخصوص کارکنان بیمارستانها به دليل تنوع در نوع افراد شاغل و نوع کاری که انجام می دهند از اهمیت ویژه ای برخوردار می باشد.این مطالعه با هدف بررسی وضعیت آسایش حرارتی کارکنان مرکز آموزشی درمانی الزهرا(س) در شهراصفهان انجام شد. روش‌ها: این مطالعه توصیفی مقطعی، جامعه مورد بررسی کلیه کارکنان  طبقه زیرزمین مرکز آموزشی درمانی الزهرا بوده است که در آنجا مشغول به کار بودند. روش نمونه گیری به صورت سرشماری و ججم نمونه 161 نفر بود. داده ها هم از طریق ارزیابی ذهنی به وسیله پرسشنامه و هم از طریق اندازه گیری فیزیکی چهارپارامتر یعنی دمای هوا، رطوبت نسبی، سرعت جریان هوا و میانگین دمای تشعشعی توسط دستگاه WBGT جمع آوری شد. شاخص PMV  به روش نرم افزاری براساس پارامترهاي اندازه گيري شده محاسبه شد و همزمان از طريق پرسشنامه نيز مورد بررسي قرار گرفت و درنهايت داده های گردآوری شده با استفاده از نرم افزار آماری SPSS20 مورد تجزیه و تحلیل قرار گرفتند.. یافته‌ها: آنالیز آماری داده های جمع آوری شده از بخش های مختلف نشان داد که بخش های آشپزخانه،لندری و استریلیزاسیون مقدار PMV به ترتيب برابر با 2.5،2.5،2.2 وشاخص PPD به ترتيب برابر 93،93 و85 بودكه از دیگر قسمتها دمای بالاتری را نشان دادند و به طور کلی به جز دفتر گروه ها و کلاس های آموزشی ديگر بخش ها و واحد هاي مربوطه در خارج از محدوده آسایش حرارتی استاندارد اشری 55 قرار داشتند نتیجه گیری: با توجه به اين كه تمام بخش ها و واحد هايي که هم از طريق ذهني و هم فيزيكي شاخص هاي آسايش حرارتي را نشان مي دادند داراي تهويه طبيعي نبودند اين نتيجه گرفته شد كه با انجام طراحی صحیح و استفاده از هوای بیرون برای تهویه طبیعی و برقراری تبادل هوا در محل کار می توان به محدوده آسایش حرارتی مطابق با استانداردهای بین المللی دست یافت.

Title: ارزیابی شدت میدان های الکترومغناطیسی کامپیوتر های همراه وارتباط آن با خستگی چشم کاربران

http://hsr.mui.ac.ir/index.php/jhsr/article/view/1175

مقدمه :با افزایش استفاده از انواع رایانه ها، میزان مواجهه با میدان های الکتریکی و مغناطیسی در حال افزایش است، مهمترین هدف از انجام این طرح بررسی میزان شدت میدان های الکتریکی و مغناطیسی در کامپیوتر های قابل حمل( لپ تاپ )های مورد استفاده و ارتباط آن با خستگی چشم کاربران بود   روش ها: در این مطالعه توصیفی– تحلیلی شدت میدان الکتریکی و مغناطیسی در فاصله  30 و 50 سانتی متری ودر چهار جهت  اصلی 125دستگاه کامپیوتر لپ تاپ اندازه گیری شد. میزان خستگی چشم دانشجویان توسط پرسشنامه ارزیابی خستگی چشم کاربران پایانه های تصویری مورد ارزیابی قرار گرفت.  یافته ها: نتایج این مطالعه نشان داد که شدت میدان  الکتریکی و مغناطیسی در 20 درصد کامپیوتر ها در فاصله 30 از بدنه کامپیوتر از حد توصیه شده بالاتر بود و در فاصله 50سانتی متری  شدت این میدان ها کمتر از حد استاندارد تعریف شده برای مانیتور ها بود.. هم چنین همبستگی معنی داری بین خستگی چشم وشدت میدان الکتریکی و مغناطیسی مغناطیسی یافت نشد.  نتیجه گیری: نتایج این پژوهش نشان داد که شدت میدان های الکتریکی و مغناطیسی در تعداد قابل توجهی از کامپیو تر های قابل حمل (لپ تاپ)، بالاتر از حد توصیه شده  است. در نتیجه به منظور کاهش میزان مواجهه با میدان های الکترومغناطیس ناشی از کار با کامپیو تر های لپ تاپ ها پیشنهاد می شود  که حداقل فاصله 50 سانتی متری بین بدن کاربر و بدنه لپ تاپ رعایت گردد

Title: طراحی و ساخت خرک ارگونومیک منطبق با مشخصات آنتروپومتریک دانشجویان دانشگاه هنر اصفهان

http://hsr.mui.ac.ir/index.php/jhsr/article/view/1259

مقدمه: شایعترین اختلالات عضلانی-اسکلتی دانشجویان کمر درد و شانه درد شناخته شده است که نحوه نشستن و تماس با تجهیزات دانشگاهی عامل اصلی بروز این ناراحتی­ها می باشد. هدف از انجام این پژوهش توسعه و ساخت مدل خرک نقاشی ارگونومی با استفاده از روش های طراحی صنعتی به منظور کاهش آسیب های عضلانی-اسکلتی و افزایش رضایت کاربران از استفاده آن می باشد. روش‌ها: بصورت یک مطالعه تجربی است که با استفاده از متدهای طراحی صنعتی و آنالیز آنتروپومتری و روش ارزیابی سریع اندام فوقانی (RULA) خرک نقاشی طراحی مجدد شده است. شرکت کنندگان 28 نفر از دانشجویان نقاشی دانشگاه هنر اصفهان می باشند، که برای طراحی و نقاشی از خرک نقاشی موجود استفاده می­کنند. یافته‌ها:14% ازشرکت کنندگان دارای امتیاز نهایی  RULA7، 39% با امتیاز نهایی 6-5 و 47% باقیمانده دارای امتیاز نهایی 3-2 بوده­اند. فاکتورهای تاثیرگذار بر امتیاز نهایی RULA جنسیت (003/0=P)، ارتفاع قد (0004/0=P) وارتفاع رکبی (004/0=P) بدست آمده است. میانگین امتیاز نهایی RULA زن­ها از مردها بیشتر بوده است. نتیجه‌گیری: ارتفاع خرک طراحی شده در پژوهش حاضر با توجه به آنالیز آنتروپومتری 430 میلیمتر تعیین شد و پشتی مناسب(با ارتفاع 330 میلیمتر) با زاویه 105 درجه نیز به طرح نهایی اضافه گردید.

Title: مواجه با دود سیگار، نور آفتاب، حیوانات خانگی و مولتیپل اسکلروزیس: مطالعه ای مورد شاهدی در اهواز( ایران)

http://hsr.mui.ac.ir/index.php/jhsr/article/view/1568

مقدمه: مطالعات بسیاری عوامل خطر ساز مربوط به سبک زندگی ابتلا به بیماری مولتیپل اسکلروزیس (MS) را مورد بررسی قرار داده اند، اما با توجه به نتایج متناقض و شرایط اقلیمی- فرهنگی ویژه شهر اهواز در ایران، این مطالعه مورد شاهدی با هدف بررسی برخی از عوامل خطر ساز مربوط به سبک زندگی (مصرف و مواجهه با دود سیگار، مدت وسطح مواجهه با نور آفتاب، مواجهه با دام یا حیوان خانگی) برای ابتلا به MS انجام گرفت. روش ها: این مطالعه از نوع مورد شاهدی دارای 155 مورد و 155 شاهد همسان سالم در انجمن MS‌ اهواز انجام شد. جهت جمع آوری داده ها، پرسشنامه سه قسمتی پژوهشگر ساخته شامل اطلاعات فردی، ویژگی های بیماری و برخی از عوامل مرتبط با سبک زندگی پس از تایید روایی و پایایی (آلفای کرونباخ 9/0) استفاده گردید. تجزیه و تحلیل آماری با استفاده از نسخه ی 19نرم افزار SPSS در سطح آمار توصیفی؛ میانگین، درصد فراوانی، انحراف معیار و  آزمون های آماری استنباطی؛ آزمون کای دو، آزمون دقیق فیشر انجام شد. یافته ها: مصرف و مواجهه با دود سیگار، سطح در معرض تماس با نور آفتاب و تماس با دام یا حیوان خانگی تاثیر معنی داری بر ابتلا به بیماری مولتیپل اسکلروزیس نداشتند، اما اختلاف معنی داری بین دو گروه از نظر مدت مواجهه با نور آفتاب و ابتلا به بیماری مولتیپل اسکلروزیس وجود داشت(04 /0 = p). نتیجه ‌گیری: این مطالعه پیشنهاد می کند که برخی عوامل محیطی نظیر مدت مواجهه با نور آفتاب می تواند در ابتلا به مولتیپل اسکلروزیس نقش داشته باشند.

Title: ارزیابی استرس گرمایی با کاربرد شاخص دمای تر گویسان ، شاخص نمره استرین گرمایی و دمای دهانی در شاغلین قنادی شهر اصفهان

http://hsr.mui.ac.ir/index.php/jhsr/article/view/1463

مقدمه: شغل قنادی از جمله مشاغلی است که تعداد قابل توجهی  از کارگران در ان اشتغال دارند و درمواجهه با   گرما قرار دارند. هدف از این مطالعه ارزیابی استرس گرمایی با کاربرد شاخص دمای تر گویسان و شاخص نمره استرین گرمایی در کارگران قنادی شهر اصفهان بود. روش ها: این مطالعه مقطعی- توصیفی در ماه های مرداد و شهریور سال 1391 بین ساعات 10 الی 14 بر روی 360 نفر از کارگران قنادی شهر اصفهان انجام شد.  برای تعیین استرس گرمایی از شاخص دمای تر گویسان یا شاخص WBGT ، برای اندازه گیری میزان استرین گرمایی درک شده توسط   کارگران از  پرسشنامه شاخص نمره استرین گرمایی یا شاخص HSSI و جهت اندازه گیری دمای دهانی از دماسنج دیجیتالی استفاده گردید . در این مطالعه عواملی نظیر فضای کارگاه، تکنولوژی پخت و وضعیت سیستم تهویه نیز مورد بررسی قرار گرفت . داده ها با نرم افزار  SPSSنسخه 16تحلیل شدند. یافته ها :میانگین شاخص WBGT محیط کار این کارگران 97/22 بود که زیر حد مراقبت (0 C28) و  حد مجاز(0 C31) برای کار سبک بود. بر اساس شاخص  HSSI ،  5/77 درصد افراد تحت مطالعه فاقد استرین گرمایی، 9/19 درصد افراد احتمالا دارای استرین گرمایی و 6/5 درصد افراد دارای استرین گرمایی بودند. نتیجه گیری: براساس نتایج این مطالعه درصد کمی از کارگران در معرض خطر استرین گرمایی بودند که می تواند  به دلیل وجود تکنولوژی  مناسب پخت ، مساحت کارگاهی مناسب  و استقرار سیستم های تهویه ی مناسب و دیگر راهکارهای کنترل گرما باشد.

Title: بررسی روش ها و انگیزه‌ها در موارد قتل های گزارش شده در تابستان 1392

http://hsr.mui.ac.ir/index.php/jhsr/article/view/1859

مقدمه: قتل یکی از هولناک‌ترین جنایت‌های بشری است که به رغم وضع قوانین مختلف برای پیشگیری از وقوع این جرم، گریبانگیر تمام جوامع بوده است. مسایل مالی، سلیقه، جنون آنی، رفتارهای جنسی نامتعارف، خشونت، دشنام و ناسزاگویی و حتی دوست داشتن و احساس تعلق از عواملی است که هر روز به عنوان انگیزه‌های قتل در حوادث روزنامه‌ها درج می‌گردد. این مطالعه برای تعیین روش‌های مختلف و انگیزه‌های احتمالی وقوع قتل گزارش شده در تابستان 92 انجام گرفته است. روش‌‌ها: این مطالعه با هدف تحلیل محتوای مطالب منتشره پیرامون قتل که در روزنامه کثیرالنتشار همشهری که در فصل تابستان 1392 منتشر شده انجام شد. تعداد کل روزنامه‌ها 81 نسخه بود و از مجموع نسخه‌های مورد بررسی، 97 مورد قتل به دست آمد. برای آنالیز اطلاعات از آمار توصیفی نظیر درصد و فروانی و از نرم افزار آماری SPSS نسخه 18 استفاده شد. یافته‌ها: بر اساس یافته‌های مطالعه 1/71 ٪ از مقتولین مذکر بودند و 6/85 ٪ قاتلین نیز مرد بودند. اکثریت قاتلین و مقتولین در رده سنی 21 تا 30 سال بود. همچنین بیشترین انگیزه قتل (8/25٪) سرقت بوده است . 34 ٪ از قتل‌ها توسط اسلحه گرم و 7/90 ٪ قتل‌ها عمدی بوده است که 7/58 ٪ از آنها با قصد قبلی انجام شده‌اند. نتیجه‌گیری: با توجه به اینکه اکثر مقتولین و قاتلین مذکر و در سنین جوانی بوده‌اند و انگیزه اصلی قتل‌ها نیز سرقت بوده است و در ضمن روش‌های مختلف قتل و انگیزه‌های آن با عوامل فردی و اجتماعی ارتباط دارد لذا توصیه می‌شود که مسؤولین جامعه اقدامات پیشگیرانه برای ارتکاب به بزه نظیر افزایش سطح رفاه جامعه، کاهش دسترسی به سلاح‌های گرم و تسریع در رسیدگی به جرایمی نظیر قتل و سرقت برای کاهش سطح ایمنی درک شده بزهکاران را اعمال نماید.

Title: ارزیابی غلظت زایلن و بررسی تاثیر پارامترهای محیطی بر آن در خودروهای ارزان قیمت تولید ایران

http://hsr.mui.ac.ir/index.php/jhsr/article/view/1082

مقدمه: زایلن به عنوان یکی از مهم‌ترین سموم دستگاه عصبی شناخته شده است. هدف از این مطالعه برآورد غلظت زایلن در کابین خودروهای تولید ایران است. همچنین چگونگی تاثیر نوع سیستم تهویه، استعمال سیگار و خوشبو کننده‌ها درون خودرو، نوع سوخت و تزئینات داخل خودرو بر روی غلظت این ماده مورد بررسی قرار گرفته است. روش‌ها: 152 خودروی پراید و 44 خودروی تندر90 از بین خودروهای ایرانی برای نمونه‌برداری انتخاب شدند. نمونه‌ها از خودروهای پارک شده در یک پارکینگ سر بسته و نیز خودروهای صفر کیلومتر در دفاتر فروش و با استفاده از تیوپ‌های کربن فعال جمع آوری شد. از دستگاه گازکروماتوگرافی با دتکتور جرمی برای آنالیز  نمونه ها استفاده شد. سایر اطلاعات مورد نیاز از جمله نوع خودرو، نوع سیستم تهویه مورد استفاده در خودرو، استعمال سیگار و خوشبوکننده ها درون خودرو، نوع سوخت و نوع تزئینات داخل خودرو با استفاده از یک چک لیست جمع آوری شدند. یافته‌ها: میانگین غلظت زایلن در خودروهای پراید و تندر 90 به ترتیب 3/1416 ± 4/657 و 06/643± 5/ 252 میکرو گرم بر متر مکعب بود. غلظت این ماده در خودروهای پراید نسبت به خودروی تندر 90 تقریبا دو برابر بود.  استفاده همزمان از بنزین و گاز طبیعی (CNG) در خودرو نسبت به استفاده بنزین به تنهایی باعث افزایش غلظت زایلن در خودروهای مورد بررسی شد. تاثیر پارامترهایی از قبیل نوع تهویه خودرو، استعمال سیگار و خوشبو کننده ها درون خودرو، تزئینات داخل خودرو تاثیر معنی‌داری بر غلظت زایلن درون خودرو نداشت. تنها نوع سوخت مورد استفاده  تاثیر معناداری بر غلظت این ماده درون خودرو داشت. نتیجه‌گیری: زایلن از جمله ترکیبات آلی فرار است که در کابین خودروهای مورد بررسی یافت شد. میانگین اندازه‌گیری شده زایلن در این مطالعه نسبت به استاندارد هونگ کونگ کمتر بود ولی نسبت به استاندارد  NIAQSکشور چین بیشتر بود.

Title: حذف تولوئن از هوا با استفاده از فرايند ازن زني كاتاليزوري

http://hsr.mui.ac.ir/index.php/jhsr/article/view/1087

مقدمه: كاربرد وسيع تولوئن در زندگي انسان و به دنبال آن اثرات زيان‌آور اين ماده بر سلامتي و به ويژه عوارض مزمن آن بر روي سيستم اعصاب مركزي، باعث گرديده است كه مطالعات زيادي در جهت كنترل و كاهش بخارات تولوئن از هوا صورت گيرد. هدف از اين پژوهش بررسي كارايي فرايند ازن زني كاتاليزوري در حذف تولوئن از جريان هوا است. روش‌ها: پژوهش حاضر از نوع تجربي و در مقياس آزمايشگاهي است. سيستم آزمايش شامل پمپ هوا، پمپ تزريق سرنگي، ژنراتور ازن، سيستم توليد رطوبت، محفظه اختلاط، روتامتر و راكتور شيشه اي به طول 25 سانتي‌متر و قطر داخلي 2 سانتي‌متر بود. غلظت‌هاي مشخص تولوئن به صورت مداوم با توجه به زمان ماند، دوز ازن و مقادير مختلف رطوبت در معرض فرايند ازن زني كاتاليزوري با کربن فعال و کربن فعال پوشش داده شده با نیترات منیزیم قرار گرفت. پس از سنجش ميزان غلظت تولوئن قبل و بعد از راكتور، داده‌ها با استفاده از نرم‌افزارSPSS  نسخه 16 و به کارگیری آمار توصيفي و استنباطي و توسط آزمون three-way ANOVA مورد تجزيه و تحليل قرار گرفت. یافته‌ها:  يافته‌ها حاكي از آن بود كه ظرفيت حذف تولوئن در فرايند ازن زني كاتاليزوري هنگام استفاده از كربن فعال پوشش داده شده با نيترات منيزيم به مراتب بيشتر از كربن فعال به تنهايي است. به طوري كه زمان ظهور تولوئن در خروجي راكتور براي كربن فعال 50 ساعت و براي كربن فعال پوشش داده شده با نيترات منيزيم 115 ساعت بود. همچنين نتايج نشان داد كه متغيرهاي زمان ماند، دوز ازن و رطوبت نسبي پارامترهاي تاثير گذار در فرايند بوده، به طوري كه افزايش سه متغير مذكور سبب ارتقا سيستم گرديد. نتيجه‌گيري: با توجه به ارتقا قابل توجه راندمان حذف تولوئن در هنگام استفاده توام از كربن فعال و نیترات منيزيم در فرايند ازن زني كاتاليزوري، مي‌توان نتيجه‌گيري كرد كه استفاده از كربن فعال پوشش داده شده با نيترات منيزيم مي‌تواند يك روش مناسب براي حذف ‌تولوئن از جريان هوا باشد.

Title: بررسی تاثیر حرکات نرمشی برنامه ریزی شده در کاهش اختلالات اسکلتی عضلانی و فرسودگی شغلی در بین کاربران کامپیوتر

http://hsr.mui.ac.ir/index.php/jhsr/article/view/1203

مقدمه: اختلالات اسکلتي- عضلاني مرتبط با کار عمده ترين عامل از دست رفتن زمان کار، افزايش هزينه ها و آسيب ها ي انساني نيروي کار به شمار مي آيند. هدف از انجام این مطالعه بررسی تاثیر استفاده از حرکات نرمشی برنامه ریزی شده در کاهش اختلالات اسکلتی عضلانی و فرسودگی شغلی در کاربران کامپیوتر بود. روش ها: در این مطالعه تاثیر حرکات نرمشی با استفاده از نرم افزار بر روی 87 نفر کاربر کامپیوتر بررسی شد. جهت بررسی اختلالات اسکلتی عضلانی و فرسودگی شغلی در ابتدا و بعد از استفاده از نرم افزار به ترتیب از پرسشنامه نوردیک و فرسودگی شغلی مزلچ استفاده شد. داده ها با استفاده از نرم افزار SPSS نسخه 21 موردتجزیه و تحلیل قرار گرفت. یافته ها: یافته های حاصل ازآمار توصیفی نشان داد که اختلالات اسکلتی- عضلانی پس از مداخله در تمام قسمت های بدن به میزان 26.43% کاهش یافته و بیشترین کاهش اختلالات نیز در ناحیه کمر با درصد فراوانی برابر 4.6% بدست آمد. نتایج حاصل از مطالعه نشان داد که اعمال مداخله بر روی کاهش اختلالات اسکلتی عضلانی در تمام بدن از نظر آماری معنی دار بود (P<0.05). هم چنین آزمونPair T-test نشان داد که متغییر فرستودگی شغلی پس از مداخله کاهش معناداری نشان نداد .(P > 0.05) نتیجه گیری: نتایج بدست آمده از مطالعه نشان داد که استفاده از حرکات نرمشی برنامه ریزی شده باعث کاهش اختلالات اسکلتی عضلانی در یک دوره شش ماهه در کاربران کامپیوتر شد. این کاهش بجز قسمت دست، در بقیه قسمت های اسکلتی عضلانی شامل گردن، شانه، کمر و … مشاهده شد. اما این مطالعه نشان داد که استفاده از حرکات نرمشی برنامه ریزی شده باعث کاهش فرسودگی شغلی و زیرمقیاس های آن در بین کاربران کامپیوتر نشد.

Title: بررسی اثر کوتاه مدت مواجهه با ارتعاش با شتاب‌های مختلف در فرکانس 7-3 هرتز بر روی توانایی های شناختی توجه

http://hsr.mui.ac.ir/index.php/jhsr/article/view/1045

مقدمه: نقص در توجه و تمركز، يكي از علل بزرگ سوانح ترافيكي محسوب مي‌شود و علت بين 20 تا 50٪ سوانح شامل شكل‌هايي از كم‌توجهي و حواس پرتي است. توجه و تمرکز به عنوان یک مولفه‌شناختی بسیار مهم در رانندگی ایمن محسوب می‌شود. همچنین مطالعات نشان داده‌اند که ارتعاش به عنوان یکی از مهم‌ترین عوامل فیزیکی در تمامی وسایل نقلیه وجود دارد. تاکنون مطالعه سازمان یافته‌ای تاثیر ارتعاش بر عملکرد شناختی خصوصاً توجه و تمرکز را مورد بررسی قرار نداده است. این مطالعه با این هدف طراحی شده است که با استفاده از آزمون‌های عصب روان‌شناختی اثر ارتعاش بر توجه و تمرکز را بسنجد. روش‌ها: در این پژوهش 25 دانشجوی مرد، در چهار مرحله شامل: شتاب کم (m/s253/0)، متوسط (m/s281/0)، زیاد (m/s212/1) و بدون مواجهه با ارتعاش (وضعیت کنترل) بر اساس استاندارد 1-2631ISO و با فرکانس ثابت 7-3 هرتز، آزمون‌های توجه انتخابی و توجه توزیع شده را درحال نشستن بر روی دستگاه شبیه ساز ارتعاش انجام دادند. آنالیز نتایج با استفاده از نرم‌افزار SPSS نسخه 19 انجام گرفت. یافته‌ها: نتایج نشان داد که اثر ارتعاش بر زمان واکنش توجه انتخابی افراد مورد مطالعه معنی‌دار نیست. ارتعاش باعث کاهش معنی‌دار تعداد پاسخ‌های صحیح توجه انتخابی و کاهش معنی‌دار زمان واکنش توجه توزیع شده می‌گردد. همچنین موجب افزایش معنی‌دار تعداد پاسخ‌های صحیح توجه توزیع شده می‌شود (05/0P≤) نتيجه‌گيری: در این مطالعه معلوم شد که ارتعاش توانایی پایداری توجه افراد مورد مطالعه را مختل می‌کند. همچنین موجب افزایش سرعت و دقت سیستم پردازش اطلاعات افراد مورد مطالعه می‌شود. به نظر می‌رسد در بلند مدت ارتعاش می‌تواند موجب خواب آلودگی فرد و عدم حفظ توجه در طول زمان رانندگی شود.

Title: بررسی رابطه فرسودگی شغلی و عملکرد بهورزان خانه های بهداشت استان آذربایجان شرقی

http://hsr.mui.ac.ir/index.php/jhsr/article/view/1034

مقدمه: سندرم فرسودگی شغلی خطرناکترین عامل تهدیدکننده سلامت شاغلین حرف بهداشتی درمانی است. این پژوهش با هدف تعیین رابطه فرسودگی شغلی و عملکرد بهورزان خانه‌های بهداشت آذربایجان شرقی انجام شد. روش‌ها: مطالعه حاضر از نوع  تحلیلی- همبستگی است که در بین 89 بهورز استان آذربایجان شرقی انجام شد. داده‌ها از طریق پرسشنامه استاندارد مازلاک و جکسون و همچنین چک لیست ارزیابی عملکرد خانه‌های بهداشت جمع‌آوری شد. داده‌ها با استفاده از نرم‌آفزار آماری SPSS ویرایش 18 و بهره‌گیری از آزمون‌های هبستگی پیرسون و تی-تست و آنوا مورد تجزیه و تحلیل قرار گرفت.. یافته‌ها: میزان فرسودگی شغلی بین بهورزان در حد متوسط و احتمال ابتلا به فرسودگی در بین آنها وجود داشت. بین فرسودگی شغلی و عملکرد و بین زیر مجموعه‌های فرسودگی شغلی شامل؛ مسخ شخصیت و تحلیل رفتگی هیجانی رابطه معنی‌دار وجود نداشت؛ ولی بین عملکرد با احساس عدم کفایت شخصی رابطه معکوس و معنی‌دار وجود داشت. نتیجه‌گیری: با اینکه در این مطالعه فرسودگی شغلی و عملکرد رابطه‌ای با هم نداشتند، لیکن نمی‌توان با قاطعیت تاثیر فرسودگی شغلی بر کارایی، اثربخشی و بهره‌وری در سازمان‌ها را رد کرد. لازم است به عوامل دیگری نیز که ممکن است بر فرسودگی شغلی یا عملکرد بهورزان تاثیر داشته باشد، توجه شود.
